# Modification of the Structural and Functional Characteristics of Mung Bean Globin Polyphenol Complexes: Exploration under Heat Treatment Conditions

**DOI:** 10.3390/foods12112091

**Published:** 2023-05-23

**Authors:** Yantao Ma, Shu Zhang, Yuchao Feng, Haoyu Wang, Yuhang Liu, Changyuan Wang

**Affiliations:** 1 College of Food, Heilongjiang Bayi Agricultural University, Xinfeng Lu 5, Daqing 163319, China; 2 National Coarse Cereals Engineering Research Centre, Daqing 163319, China; 3 Heilongjiang Food and Biotechnology Innovation and Research Center (International Cooperation), Daqing 163319, China

**Keywords:** mung beans, polyphenols, heat processing, interactions, antioxidant activity

## Abstract

During the storage and processing of mung beans, proteins and polyphenols are highly susceptible to interactions with each other. Using globulin extracted from mung beans as the raw material, the study combined it with ferulic acid (FA; phenolic acid) and vitexin (flavonoid). Physical and chemical indicators were combined with spectroscopy and kinetic methods, relying on SPSS and peak fit data to statistically analyze the conformational and antioxidant activity changes of mung bean globulin and two polyphenol complexes before and after heat treatment and clarify the differences and the interaction mechanism between globulin and the two polyphenols. The results showed that, with the increase in polyphenol concentration, the antioxidant activity of the two compounds increased significantly. In addition, the antioxidant activity of the mung bean globulin–FA complex was stronger. However, after heat treatment, the antioxidant activity of the two compounds decreased significantly. The interaction mechanism of the mung bean globulin–FA/vitexin complex was static quenching, and heat treatment accelerated the occurrence of the quenching phenomenon. Mung bean globulin and two polyphenols were combined through a hydrophobic interaction. However, after heat treatment, the binding mode with vitexin changed to an electrostatic interaction. The infrared characteristic absorption peaks of the two compounds shifted to different degrees, and new peaks appeared in the areas of 827 cm^−1^, 1332 cm^−1^, and 812 cm^−1^. Following the interaction between mung bean globulin and FA/vitexin, the particle size decreased, the absolute value of zeta potential increased, and the surface hydrophobicity decreased. After heat treatment, the particle size and zeta potential of the two composites decreased significantly, and the surface hydrophobicity and stability increased significantly. The antioxidation and thermal stability of the mung bean globulin–FA were better than those of the mung bean globulin–vitexin complex. This study aimed to provide a theoretical reference for the protein–polyphenol interaction mechanism and a theoretical basis for the research and development of mung bean functional foods.

## 1. Introduction

As an important food component, protein is valued for its unique nutritional and functional characteristics. Additionally, it plays a role in emulsification, foaming, and other functions in the food system [1,2]. However, its antioxidant activity is poor [3]. Protein interaction with other components in food (such as phenolic compounds) is a promising method for preparing novel protein-based compounds with enhanced antioxidant activity [4,5]. Polyphenols are widely used in food, medicine, and other fields because of their unique structure and functional characteristics. Proteins and polyphenols, two common nutrients in food, can easily interact during food storage, transportation, and processing, affecting their structural, functional, and nutritional characteristics. As a common processing method, heat treatment can affect the interaction of components in the complex, induce conformational changes, and change the nutritional quality of the entire system [6]. Because of their unique structures and biological activity, polyphenol protein complexes can be prepared as functional lotions, liposomes, microcapsules, nanoparticles, and other new complexes, and they can also be used as stabilizers of metal nanoparticles, demonstrating their prospective applications in the fields of food and medicine [7]. For example, Zhao et al. [8] prepared gallic acid zein nanoparticles and used them as an interfacing stabilizer for Pickering lotion droplets. Hu et al. [9] observed for the first time that when high concentrations of amyloid fibrils were present, small molecular compounds of polyphenols could be adsorbed to the surface of globulin through hydrophobic interaction, π–π conjugation, and hydrogen bonding. This drove the self-assembly of nanofiber supramolecules in the liquid crystal state, forming a three-dimensional reticular multi-level structure hydrogel filled with water in space, and the drug load of polyphenols significantly increased (the mass fraction reached 4%). The stability has been significantly improved, and it has a broad spectrum of antibacterial effects. Studies have shown that proteins and polyphenols are either covalently (e.g., via free radical grafting, alkali treatment, and enzymatic catalysis) or noncovalently bound, and most of the protein–plant polyphenols found in nature exist through non-covalent binding forms. The interaction between the two components usually reduces or enhances the antioxidant activity of phenolic substances while altering the protein structure [10,11]. The interaction is affected by environmental conditions and the types of structures involved, which leads to changes in the functional and nutritional properties of the complex [12,13,14]. For example, the interaction between chlorogenic acid and whey protein enhances the antioxidant activity of the complex [15], while the interaction between chlorogenic acid and bovine serum protein leads to the decline of the antioxidant activity of the complex [16]. The interaction of proteins and polyphenols can broaden their prospective applications, providing complexes with novel characteristics.

Mung bean is a type of coarse grain crop with uses in both medicine and food; it is rich in nutrients and bioactive substances. Mung bean protein contains various amino acids that are essential for the human body. The 8S globulin is structurally similar to the β-soybean globulin (7S globulin) [17] and has good solubility, emulsification, and stability. As a source of high-quality protein, mung beans have great potential in the development of plant-based dairy products [18]. In addition, mung bean reduces blood sugar and has heat-clearing, detoxing, anticancer, and liver protection effects [19,20], which are mainly attributed to its polyphenols [21]. Vitexin and ferulic acid (FA) are important as the mung bean’s representative flavonoids and phenolic acids [22]. Vitexin has antibacterial, antioxidant, and cardiac protection functions and is widely used in the prevention and treatment of diseases [23,24]. FA is an antioxidant with anti-inflammatory, antibacterial, antimutation, and anticancer properties. Additionally, it has shown considerable potential in domestic and foreign markets, and many countries have begun to apply it to various fields [25]. Previously, we explored the interaction between mung bean globulin and mung bean total phenols and observed that adding mung bean total phenols changed the structure and functional activity of mung bean globulin. However, mung bean polyphenols are complex and contain various monomer phenols; different polyphenols can play a synergistic or antagonistic role in the complex system. Therefore, it was impossible to judge the structure–activity relationship between mung bean polyphenols and mung bean globulin and the change in antioxidant activity from mung bean total phenols, judging the structure–activity relationship between mung bean polyphenols and mung bean globulin and the change in antioxidant activity from mung bean total phenols was impossible.

Guo [26] explored the interaction between mung bean protein and vitexin and reported that there were differences between the structure and activity of the mung bean protein–vitexin and mung bean globulin–mung bean polyphenol complexes, because the mung bean protein in the protein–vitexin complex comprised a mixture of various proteins. Moreover, it was impossible to determine the structure–activity relationship between the two; the binding site of the complex and the phenolic hydroxy sites played an antioxidant role in the total mung bean protein, and the interaction between different types of polyphenols and mung bean globulin differed. Therefore, in this study, the flavonoid vitexin and phenolic acid FA in mung bean were combined with mung bean globulin. The possible interaction between the two compounds in the mung bean system was simulated, and the differences between the structure and antioxidant activity of the two compounds were clarified. The effects of heat treatment on the structures and antioxidant activities of the two compounds were explored to provide a theoretical basis for the interaction in the food system and the development of mung bean functional food.

## 2. Materials and Methods

### 2.1. Materials

Mung bean (Ming mung bean) was obtained from Shanxi Dongfang Liang Life Science and Technology Co., Ltd. (Datong, China). Sodium chloride, hydrochloric acid (HCl, 12 mol/L), sodium hydroxide (NaOH, 96%), trichloroacetic acid, FA, and vitexin were obtained from Shanghai Yuanyuan Biological Co., Ltd. (Shanghai, China). Finally, the 1,1-Diphenyl-2-trinitrophenylhydrazine (DPPH) kit, the 2,2′-azino-bis (3-ethylbenzothiazoline-6-sulfonic acid) (ABTS) kit, and the Total Antioxidant Capacity Assay Kit with the FRAP method (PRAP) kit were obtained from Biyuntian Biotechnology Co., Ltd. (Shanghai, China).

### 2.2. Instruments

The following instruments were used: fluoromax-4 fluorescence spectrometer (HITACHI, Tokyo, Japan); SevenEasy pH metre and EL 204 electronic balance (Mettler–Toldo Instruments Co., Ltd., Shanghai, China); frozen centrifuge (Sigma–Aldrich Company, St. Louis, MO, USA); and UV 5300PC UV–Visible Spectrophotometer (Shanghai Analysis Instrument Co., Ltd., Shanghai, China).

### 2.3. Preparation of Mung Bean Globulin

Referring to the method of Zhang et al. [27], the extraction of mung bean globulin was as follows: 0.5 mol/L acidified salt solution (containing 0.025 mol/L hydrochloric acid) was added to the mung bean powder (10.0%, *w*/*v*) and stirred for 2 h at 25 °C to keep the pH value stable. The suspension was centrifuged at 12,000× *g* for 20 min in a cold centrifuge, and the resulting supernatant was diluted with triple the volume of distilled water and placed in a cold bath for 30 min. After centrifuging the diluted supernatants twice (12,000× *g*, 20 min) at 4 °C, the precipitates were then collected. Finally, the precipitates were dialyzed in distilled water at 4 °C for 48 h and then lyophilized to obtain globin-rich mung bean protein samples.

### 2.4. Protein Purity Determination

The Kjeldahl nitrogen determination method was used. Refer to Zhang’s method and select appropriate conditions for determination [28]: weigh 1 g of protein sample in a conical flask, add 0.5 g Cu_2_SO_4_, 10 g KSO_4,_ and 20 mL of concentrated sulfuric acid, shake well and heat to make the liquid in the flask boil slightly continuously. When the color of the solution changed to clear blue–green solution, the solution was treated at constant temperature for 30 min and then cooled and set aside in a 100-mL volumetric flask. The sample was distilled using a semi-automatic Kjeldahl nitrogen tester after cooling and titrated with 0.01 mol/L hydrochloric acid standard solution after distillation, and the titration was stopped when the color of the solution changed to slightly red and the amount of hydrochloric acid used was recorded. The mass fraction of protein was calculated according to the formula:

(1) $$W=\frac{c\times \left(V_{2}-V_{1}\right)\times 0.014\times F}{m\times \frac{10}{100}}\times 100\%$$

W: mass fraction of protein, %; c: concentration of hydrochloric acid standard solution, mol/L. V_1_: the amount of standard solution consumed for blank titration, mL. V_2_: the amount of standard solution consumed for the titration of reagents, mL. m: mass of sample, g. 0.014: millimolar mass of nitrogen; F: Protein coefficient.

### 2.5. Preparation of the Protein–Polyphenol Complex

The mung bean protein solution was diluted to 1 g/100 mL with a phosphate buffer solution (PBS) (0.1 mol/L, pH = 7.0). Mung bean polyphenols were prepared with PBS solution of the same concentration (ready for use). The final concentrations of polyphenol were 0, 40, 60, 80, 100, and 120 μmol/g pro and 0, 20, 40, 60, 80, and 100 μmol/g pro. After reacting at 25 °C for 2 h, the solution was adjusted to the isoelectric point of mung bean protein (pH = 4.6) and then centrifuged. After the experimental screening, the effect of heat treatment on the composite was explored further at a fixed ratio. According to the research of Qiao [29], the denaturation temperature of mung bean globulin is 76.46 °C. Therefore, the following heat treatment conditions were used in this study: 25 °C (room temperature), 70 °C (without protein denaturation), 80 °C and 90 °C (with complete denaturation), 100 °C (with a common processing temperature).

### 2.6. Protein–Polyphenol Binding Rate and Mass Ratio

Determination by reference to Zhang’s method [27]. The content of mung bean polyphenols in the supernatant discarded during the preparation of the compound was determined by the folinol method:

(2) $$\mathrm{Polyphenol}\;\mathrm{binding}\;\mathrm{rate}\left(\%\right)=\frac{\mathrm{Polyphenol}\;\mathrm{content}-\mathrm{content}\;\mathrm{of}\;\mathrm{polyphenols}\;\mathrm{in}\;\mathrm{supernatant}}{\mathrm{Polyphenol}\;\mathrm{content}}$$

(3) $$\mathrm{Polyphenol}-\mathrm{protein}\;\mathrm{mass}\;\mathrm{ratio}\left(\%,w/w\right)=\frac{\mathrm{Polyphenol}\;\mathrm{content}\left(\mathrm{mg}\right)\times \mathrm{Polyphenol}\;\mathrm{binding}\;\mathrm{rate}\left(\%\right)}{\mathrm{Quality}\;\mathrm{of}\;\mathrm{protein}\left(\mathrm{mg}\right)}$$

### 2.7. Solubility Properties

Bovine serum protein was used as the standard, and the standard curve equation was y = 0.2024x + 0.0003 (R^2^ = 0.9975). The solubility of the composite was determined by the method of Zhang et al. [27]. The solubility of the protein was calculated according to Equation (4).

(4) $$\mathrm{Solubility}\left(\%\right)=\frac{\mathrm{Soluble}\;\mathrm{protein}\;\mathrm{content}\left(g\right)}{\mathrm{Protein}\;\mathrm{content}\;\mathrm{of}\;\mathrm{sample}\left(g\right)}\times 100\%\;$$

### 2.8. Determination of DPPH Free Radical Scavenging Activity

The method for determining DPPH free radical scavenging activity was slightly modified with reference to Wu et al. [30]. First, the DPPH working solution was diluted with ethanol to an absorbance of 0.70 ± 0.02. Next, the solution was kept in the dark for 30 min, absolute ethanol was used as the blank reference, and an absorbance value of 515 nm was measured. The calculation formula was as follows:

(5) $$\mathrm{DPPH}(\%)=(1-\frac{A_{1}-A_{3}}{A_{2}})\times 100\%$$

A_1_: 1 mL solution to be tested + 2 mL DPPH; A_2_: 1 mL absolute ethanol + 2 mL DPPH; A_3_: 1 mL solution to be tested + 2 mL absolute ethanol.

### 2.9. Determination of ABTS+ Free Radical Scavenging Activity

The method of Lahouar et al. [31] was slightly modified to determine the ABTS+ free radical scavenging activity. The prepared ABTS+ stock solution was incubated overnight in the dark and diluted with ethanol before use to produce a working solution with an absorbance of 0.70 ± 0.02 at 734 nm. A 0.2 mL of sample solution and 2.0 mL of freshly prepared ABTS+ free radical working solution were mixed, and the absorbance standard at 734 nm (A_X_) was measured. The results are expressed as percentage activity (%):

(6) $$\mathrm{ABTS}+(\%)=(1-\frac{A_{X}-A_{X0}}{A_{X}})\times 100\%$$

A_x0_ was 0.2 mL of sample solution + 2 mL of solvent; A_0_ was 0.2 mL solvent + 2 mL ABTS+ working solution.

### 2.10. Ferric-Reducing Antioxidant Power

The assay was performed with reference to the method of Li et al. [32]. The FRAP working solution consisted of 300 mmol/L acetate buffer (pH 3.6), 100 mmol/L TPTZ hydrochloric acid solution and 20 mmol/L FeCl_3_ mixed at 10:1:1 (*v*:*v*:*v*), ready to use, and preheated in a water bath at 37 °C before use. The standard curve was plotted with the mass concentration of FeSO_4_–7H_2_O as the horizontal coordinate and the absorbance value at 593 nm as the vertical coordinate. The standard curve was obtained as y = 0.2433x + 0.0221. 0.2 mL of sample was mixed with 3 mL of FRAP working solution, and the 593 nm absorbance value ($A_{0}^{\prime }$) was measured for 30 min by avoiding light; 0.2 mL of acetone and methanol mixture (7:2/*v*:*v*) were mixed with 3 mL of FRAP working solution, and the 593 nm absorbance value ($A_{0}^{\prime }$) was also measured for 30 min by avoiding light. The results were expressed as μmol/L Fe^2+^ per mg of dried sample (μmol/L Fe^2+^/mg DW) by substituting ($A_{0}^{\prime }$–A_0_) into the standard curve.

### 2.11. Fluorescence Spectrometry

A FluoroMax–4CP fluorescence spectrometer was used to record fluorescence dynamics. Fluorescence tests were carried out under constant temperature conditions using water baths at 25 °C, 70 °C, 85 °C, 90 °C, and 100 °C. The protein concentration was fixed at 1 g/L. The prepared mung bean polyphenol solution was added and mixed well. Fluorescence scanning was performed after a 2-h immersion in the water bath. Fluorescence spectral determination conditions were as follows: excitation wavelength = 240 nm; emission wavelength = 300–450 nm; and excitation and emission slit widths = 5 nm. The measuring conditions for two- and three-dimensional spectra were identical [33].

### 2.12. Fluorescence Quenching Mechanism

The resulting spectral data were further analysed using the Stern–Volmer equation to clarify the quenching mechanism of the interaction between mung bean protein and polyphenols. According to the equations of Hill and Van’t Hoff, [Q] was used as the independent variable and F_0_/F as the dependent variable, thus calculating the K_SV_ and K_q_ parameters for the interaction between mung bean polyphenols and protein as follows:

(7) $$\frac{F_{0}}{F}=1+Kq\tau_{0}[Q]=1+K_{\mathrm{SV}}[Q]$$

F_0_ represented the fluorescence intensity of the initial mung bean protein; F was the fluorescence intensity of the mung bean protein after adding a fluorescence quenching agent (mung bean polyphenol); K_q_ was the dynamic quenching rate constant of protein; τ_0_ was the fluorescence lifetime of biological macromolecules in the absence of mung bean polyphenols, which was approximately 10^−8^ S^−1^ [34]; K_SV_ was the dynamic quenching constant; and [Q] was the concentration of the quenching agent mung bean polyphenol (mol/L).

### 2.13. Calculation of the Binding Constant and Binding Site

Linear fitting was performed using the Lineweaver–Burk equation with lg[Q] as the independent variable and lg[(F_0_ − F)/F] as the dependent variable to calculate the binding constant K_A_ and binding site n (it was predicted that there were n independent and identical binding sites on mung bean protein).

(8) $$\mathrm{lg}\left[\frac{F_{0}-F}{F}\right]=\mathrm{lg}K_{A}+n\mathrm{lg}[Q]$$

### 2.14. Thermodynamic Parameters and Force Type

According to the enthalpy (∆H) and entropy (∆S), the interaction model of a small molecule active substance and a large molecule protein could be obtained. The following equation was used to calculate this:

∆G = −RTlnK_A_ (9)

(10) $$\mathrm{Ln}\frac{K2}{K1}=\frac{∆H}{R}\;\times \;(\frac{1}{T1}-\frac{1}{T2})$$

∆G = ∆H − *T*∆S (11)

R was the molar gas constant, 8.314 J/(mol·K); *T* was the temperature specified for the experiment.

### 2.15. Fourier Infrared Spectrum Analysis

The infrared spectrum of mung bean protein was recorded at 25 °C using the TENSOR II Fourier transform infrared spectrometer (Bruker, Billerica, MA, USA). The sample was mixed and ground with KBr powder and pressed to form a tablet (mass ratio 1:100). Full band scanning was conducted between 400 and 4000 cm^−1^ [35].

### 2.16. SDS–PAGE Polyacrylamide Gel Electrophoresis

The sample was dissolved in a 0.1 mol/L NaOH solution to produce a concentration of 0.5 mg/mL. An identical amount of sample loading buffer was added and mixed well, then placed in a water bath at 100 °C for 5 min to denature the protein. Electrophoresis was conducted with 5% concentrated gel, 12% separated gel, and 10 μL of sample loading buffer. Pressurising ceased when the strip ran to the bottom of the rubber. After 2 h of dyeing, the samples were eluted with the decolourizing solution for 2 h, which was repeated three or four times [28].

### 2.17. Particle Size Distribution and ζ Potential

The mung bean protein–polyphenol complex was determined using a particle size analyzer, and a 0.5 mg/mL sample solution was prepared with PBS buffer (50 mmol/L, pH 7.0) at 25 °C [36].

### 2.18. Determination of Surface Hydrophobicity

The surface hydrophobicity of the composite was determined using the ANS method [35]. The sample was dissolved in PBS (pH = 7, 0.01 mol/L) to a concentration of 1 mg/mL. After centrifugation, the protein concentration of the supernatant was measured and diluted. The concentration gradient diluent was mixed with 8 mmol/L ANS in a volume ratio of 100:1. After 3 min, the fluorescence intensity of the solution was measured, with an excitation wavelength of 390 nm and an emission wavelength of 468 nm. The sample surface hydrophobic value, H0, was determined from the gradient of the line fitting the fluorescence intensity (the dependent variable) to the concentration of the mung bean protein–polyphenol complex (the independent variable).

### 2.19. Data Statistics and Analysis

The experiment was repeated three times, and the results were expressed as the mean ± standard deviation. The data were processed using Sigmaplot12.5 (San Jose, CA, USA) and Origin 2023 software (Northampton, MA, USA) for mapping, and the structure analysis was performed using PeakFit 4.12 software (San Jose, CA, USA). The statistical analysis was performed using single factor analysis of variance (ANOVA) and the Duncan test (*p* < 0.05) using IBM SPSS 21 software (Armonk, NY, USA).

## 3. Results and Analysis

### 3.1. Binding Amount

**Figure 1:**
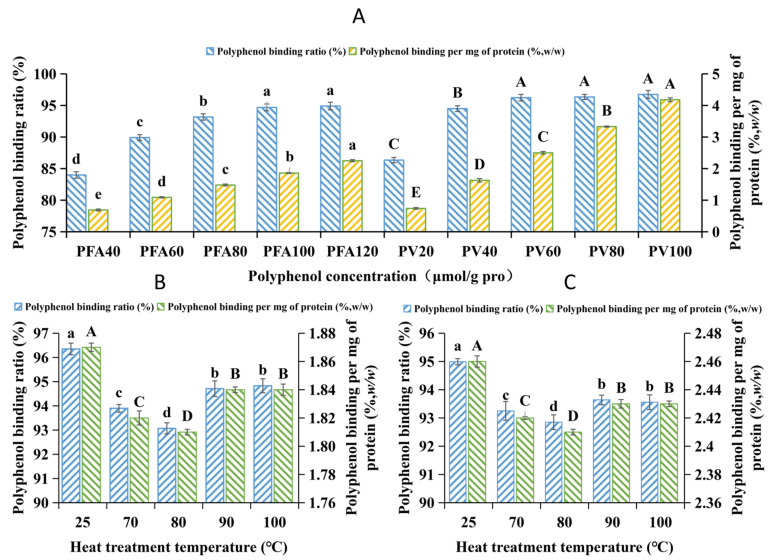
(**A**) The binding rate and mass ratio of mung bean globulin to vitexin (20, 40, 60, 80, and 100 μmol/g pro) and ferulic acid (FA) (40, 60, 80, 100, and 120 μmol/g pro) at different ratios; (**B**) Binding rate and mass ratio of mung bean globulin and FA at of 25, 70, 85, and 100 °C; (**C**) Binding rate and mass ratio of mung bean globulin and vitexin at 25, 70, 85, and 100 °C. Note: Different letters indicate differences. There were significant differences between the groups (*p* < 0.05).

Mung bean globulin with a purity of 87.3% was combined with two polyphenols. The binding of mung bean globulin with FA/vitexin is shown in Figure 1. With the increase in the concentration of FA and vitexin, their binding rate and mass ratio to globulin were significantly increased. There was a higher binding rate between vitexin and mung bean globulin, which might be related to the structural difference between the two polyphenols, and this was consistent with the results of Bohin et al. [37]. The quantity of the phenolic hydroxyl group of vitexin was greater than that of FA, which made it easier to bind with mung bean globulin. The binding rates and mass ratios of proteins and the two monomeric phenols were significantly decreased. The trends of binding rate and mass ratio were exactly the same. After reaching 90 °C, polyphenols tend to react with protein components with a large molecular weight [38], and the protein molecules are degraded after heat treatment. However, the effect of continuous heating after reaching 90 °C on the two indices was not significant.

### 3.2. Antioxidant Activity

**Figure 2:**
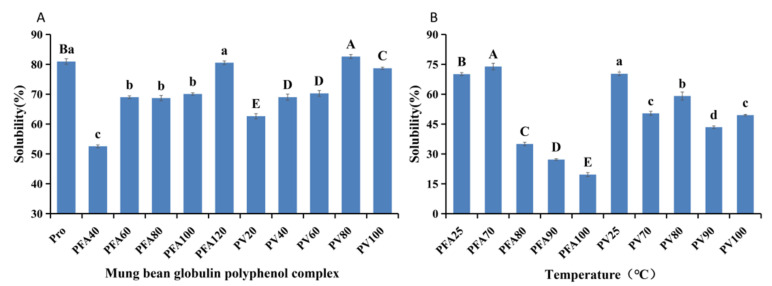
(**A**) solubility of the complexes at different ratios; (**B**) solubility of the complexes at different temperatures. The upper case letters in Figure (**A**) show the significance between the pv groups and the lower case letters show the difference between the PFA groups; the lower case letters in Figure (**B**) show the significance between the pv groups and the upper case letters show the difference between the PFA groups.

**Figure 3:**
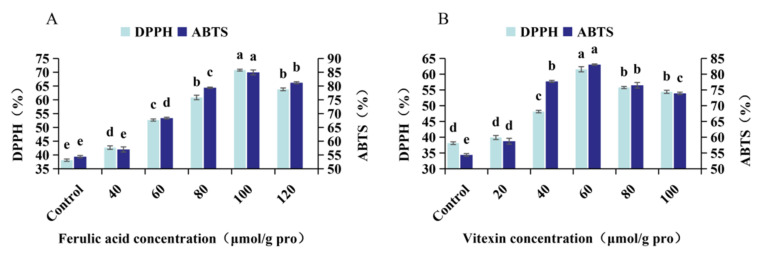
(**A**) ABTS+ and DPPH of the mung bean globulin–ferulic acid complex in different proportions; (**B**) ABTS+ and DPPH of the mung bean globulin–vitexin complex at different proportions. Different letters within a column indicate significant differences for each parameter (*p* < 0.05).

The solubility of the mung bean globulin polyphenol complex is shown in Figure 2. With the addition of polyphenols, the solubility of the complex showed an increasing trend but decreased compared with that of mung bean globulin. However, in the subsequent antioxidant study, we used a sample mass of 8 mg/mL and a phosphate buffer concentration of 0.1 mol/L, pH 8.4, under which the complexes could be completely soluble. We used Vc as the control group, and the free radical scavenging activity of Vc measured under the same conditions was 96.13%. DPPH and ABTS were used to compare the antioxidant capacity of the mung bean globulin–polyphenol complex at different interaction ratios (Figure 3). The antioxidant activity of the complex first increased and then decreased with the increase in the FA and vitexin concentrations. When the FA and vitexin concentrations reached 100 and 60 μmol/g pro, respectively, the antioxidant capacities of the two compounds reached their respective maximums. After the interaction between mung bean globulin and the two phenol monomers, the functional groups with antioxidant capacity on the molecule of the complex were exposed, thus increasing the antioxidant capacity. When the polyphenol concentration continued to increase, it was difficult for many polyphenol molecules to find binding sites on the mung bean globulin molecule. Excessive polyphenols changed the charge number of the protein molecule surface; therefore, the mung bean globulin–polyphenol complex aggregated and precipitated, and the functional groups with antioxidant ability were not exposed, resulting in a decreased antioxidant ability [39]. At the same concentration, the antioxidant activity of the mung bean globulin–FA complex (PFA) was significantly greater than that of the mung bean globulin–vitexin complex (PV). This activity may be because the 4′ phenolic light group on the B ring of vitexin was the first active site for scavenging free radicals. During the interaction between protein and vitexin, the protein combined with this site and covered it up, resulting in a low antioxidant capacity [40]. Zhang et al. reported that the mung bean globulin polyphenol complex can significantly alleviate oxidative stress levels in ageing mice, resulting in delayed ageing [41]. Therefore, the FA and vitexin concentrations were 100 and 60 μmol/g pro, respectively, for subsequent experiments. With increasing temperature, the antioxidant capacity of the two compounds decreased to varying degrees (Table 1), because, when the temperature was high, the complex structure of the polyphenol protein became uncoupled, and certain polyphenols were freed from the binding state in the complex [42]. In addition, during the heating process, certain polyphenols were converted into quinones and covalently bound to proteins, which reduced the antioxidant capacity of the system [43,44]. Chi et al. reported that the antioxidant capacity of a soy protein–polyphenol complex also decreased after heating, which was consistent with the results of this study [45].

### 3.3. Fluorescence Spectrum

**Figure 4:**
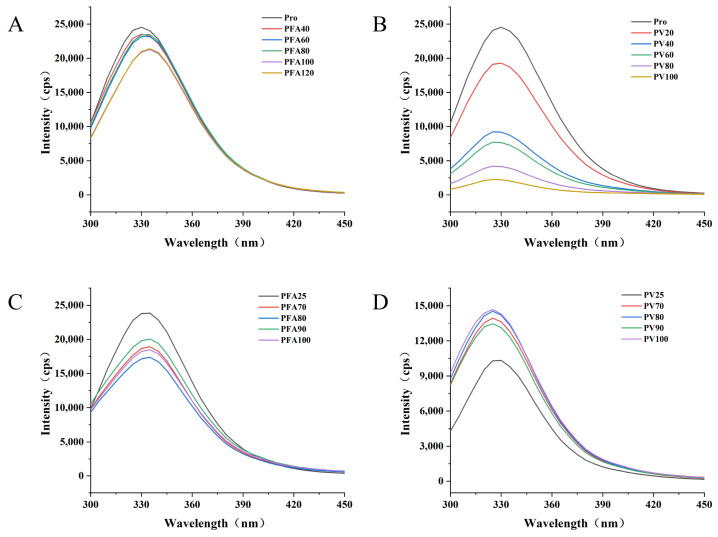
Two-dimensional fluorescence spectrum: (**A**) Mung bean globulin–ferulic acid complex in different proportions; (**B**) Mung bean globulin–vitexin complex in different proportions; (**C**) Mung bean globulin–ferulic acid complex at different temperatures; (**D**) Mung bean globulin–vitexin complex at different temperatures.

**Figure 5:**
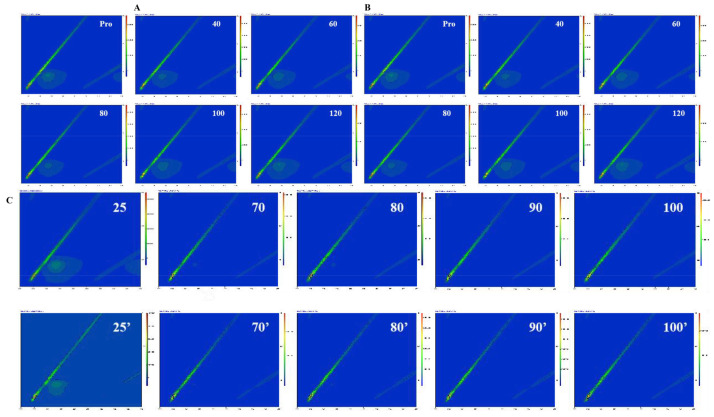
Three-dimensional fluorescence spectrum: (**A**) Mung bean globulin–ferulic acid complex in different proportions; (**B**) Mung bean globulin–vitexin complex in different proportions; (**C**) Effect of temperature on the mung bean globulin–polyphenol complex; 25–100 represent mung bean globulin–ferulic acid complexes, 25′–100′ represent mung bean globulin–vitexin complexes.

The endogenous fluorescence of proteins is due to the existence of tryptophan (Trp) residues, and the relative position of residues can be reflected by the fluorescence peak position. A high fluorescence intensity indicates that the protein is in a folded state, and the Trp residue is typically located in a hydrophobic environment [46]. When the contents of two polyphenols in the composite system gradually increase, the endogenous fluorescence intensity of mung bean globulin gradually decreases (Figure 4). By adding FA, the maximum emission wavelength of mung bean globulin shifted red, which showed an interaction between them. The interactions may be due to the fluorescence quenching between mung bean globulin and FA; the tertiary structure of the protein molecule stretched, and simultaneously, the aromatic ring of polyphenols combined with the tyrosine residues of mung bean globulin. The hydrophobicity of the microenvironment of the aromatic amino acid residues decreased, the polarity increased, and the spatial structure of mung bean globulin gradually became more extended [47], which caused the endogenous fluorescence quenching of mung bean globulin. However, with the addition of vitexin, the maximum fluorescence peak of globulin blue shifted from 330 to 325 nm, which showed that vitexin decreased the polarity of the microenvironment in which Trp residues in mung bean globulin are located, increased the hydrophobicity and decreased the hydrophilicity, which caused the conformation change in mung bean globulin. In addition, compared with the two different polyphenols, the decrease in globin fluorescence intensity was greater than that of FA, which may be due to the higher molecular weight of vitexin and the stronger affinity with mung bean globulin; therefore, the binding strength of PV was greater than that of PFA. Yu confirmed that different types and concentrations of polyphenols have different effects on protein fluorescence quenching [48]. Although the maximum peak position of PFA did not change after heat treatment, its fluorescence intensity value decreased significantly. In addition, the fluorescence emission peak shape of PV did not change significantly, the fluorescence intensity at the maximum absorption wavelength decreased significantly, the maximum emission wavelength of the complex moved from 330 to 325 nm, and a blue shift occurred. Mung bean globulin molecules gathered Trp, and hydrophobicity was weakened. This indicated that the increase in temperature could further affect the interaction between mung bean globulin and the two polyphenols. Three-dimensional fluorescence spectra showed that the fluorescence intensity of the complex was negatively correlated with the number of polyphenols added, and that the temperature accelerated the fluorescence quenching of the complex (Figure 5). This was consistent with the results of Zhang et al. [27].

### 3.4. Quenching Mechanism

**Figure 6:**
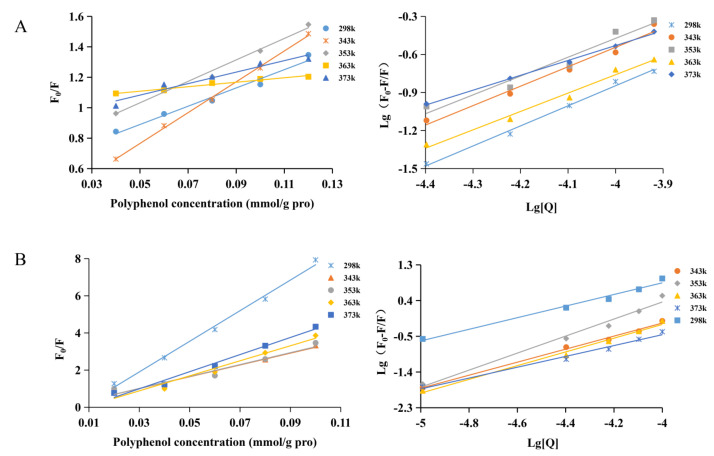
Stern–Volmer equation and double logarithmic diagram of the interaction between mung bean globulin and polyphenols at 298 K (25 °C), 343 K (70 °C), 353 K (80 °C), 363 K (90 °C), and 373 K (100 °C): (**A**) PFA; (**B**) PV.

When protein and polyphenol are used as a fluorescent group and quenching agent, respectively, the fluorescence quenching of protein can be divided into two types. The first is static quenching, which forms non-fluorescent complexes between proteins and polyphenols. The other is dynamic quenching, which is fluorescence quenching due to dynamic collisions between proteins and polyphenols. In general, dynamic quenching only affects the excited state of quenched molecules. However, it does not change the absorption spectrum of quenched substances [49]. According to the Stern–Volmer equation, the endogenous light quenching curve of polyphenols on mung bean globulin was obtained with [Q] and F_0_/F as the independent and dependent variables, respectively. The dynamic quenching constant and bimolecular quenching rate constant of the globulin–polyphenol complex system were calculated from the slope of the line (Figure 6), and the results are shown in Table 2. At different temperatures, the binding constant of PFA/PV decreased with the increase in temperature, and the quenching rate constant K_q_ was substantially greater than 2 × 10^10^ L/mol·s, indicating that its quenching type was static. The quenching constant of PV was greater than that of PFA, indicating that the stability of PV was stronger than that of PFA. This was consistent with the above results. The n of the two complexes was >1, indicating that there was a binding site between the two polyphenols and globulin. Zhang et al. reported three binding sites between mung bean globulin and total phenols [27]. This might be because the total phenols of mung bean were a mixture of multiple phenolic substances and contained different types of polyphenols; the higher the flexibility of the polyphenol molecules and the freer the gallic acid groups, the more binding sites and the stronger the affinity. The results of this experiment were consistent with those of Guo [26]. Wang reported that globulin was the main protein in mung bean [22]; therefore, it was concluded that globulin was the main component of mung bean protein interacting with vitexin.

The interaction between mung bean polyphenols and globulin molecules was investigated according to Van‘t Hoff’s formula and law. The relevant thermodynamic parameters were calculated, and the results are shown in Table 2. ΔH > 0 (enthalpy) and ΔS > 0 (entropy) represent hydrophobic interaction; ΔH < 0 and ΔS < 0 represent van der Waals forces and hydrogen bonds, and ΔH < 0 and ΔS > 0 indicate electrostatic interaction. During the binding process of mung bean globulin and FA, ΔH > 0, ΔS > 0, and ΔG < 0 indicated that the combination of the two was a spontaneous process, their interaction was mainly non-covalent, the leading role was a hydrophobic interaction, and the combination process was an entropy-driven reaction. However, PV was slightly different from PFA. The results showed that the binding process of mung bean globulin and vitexin was ΔS > 0, ΔG < 0. However, at 70 °C and 80 °C, ΔH < 0 indicated that the interaction between the two was mainly electrostatic. The main interaction between them was hydrophobic when the temperature reached 90 °C and 100 °C. The difference between the two forces in the early stage might be related to their structures. Vitexin had more hydroxyl groups, and the number of benzene rings in vitexin was greater than that of FA, which led to the stronger steric hindrance effect of vitexin [32]. The hydrophobic interaction was a strong force formed between non-polar groups; the hydrophobic amino acid residue of mung bean globulin interacted with the non-polar aromatic ring of vitexin [11], and the site on the benzene ring of vitexin was occupied. Therefore, a hydrophobic interaction formed more easily with FA than with vitexin. After heat treatment, the structure of vitexin changed, and the interaction between mung bean globulin and vitexin became hydrophobic. The types of proteins and polyphenols involved in an interaction affect their binding ability. Wang used fluorescence spectroscopy and Fourier transform infrared spectroscopy to study the interaction between anthocyanins in black bean skin and wheat proteins melololin and glutenin under neutral conditions [50]. The results showed that anthocyanins and melololin were mainly bound through hydrophobic interactions, whereas anthocyanins and glutenin were bound by van der Waals forces and hydrogen bonds. Gao et al. [51] reported that zein binds with tannic acid and EGCG through hydrogen bonding, while zein binds with gallic acid to form a complex through hydrophobic interactions. Therefore, the results obtained in this study are verified.

### 3.5. Fourier Transform Infrared Spectroscopy

**Figure 7:**
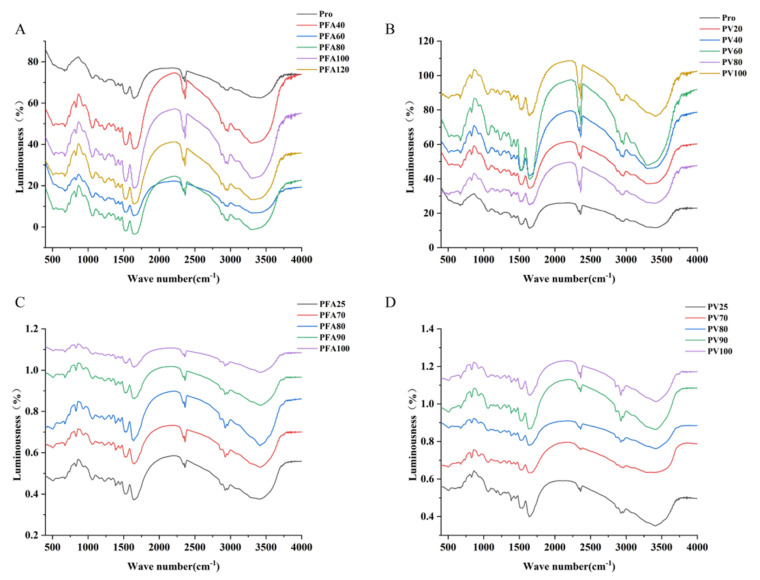
Fourier infrared spectroscopy. (**A**,**B**) Mung bean globulin–ferulic acid complex and mung bean globulin–vitexin complex at different ratios; (**C**,**D**) Mung bean globulin–Ferulic acid complex and mung bean globulin–vitexin complex at different temperatures.

The two most significant vibrational bands of a protein skeleton are the amide I (1600–1700 cm^−1^) and II (1480–1580 cm^−1^) bands. The Amide I band is the most sensitive spectral region to the secondary structural components of proteins and is due to the C=O tensile vibration of peptide bonds. Amide II mainly comes from in-plane -NH bending and -CN tensile vibration [52]. With the addition of FA/vitexin, PV and PFA shifted 2 cm^−1^ and 1 cm^−1^ in the amide I band, respectively, and the results showed that the addition of FA/vitexin changed the secondary structure of mung bean globulin, broke the intramolecular chemical bond of mung bean globulin, caused the association reaction of the N–H bond, increased the interaction between protein molecules, and stabilized the structure of the complex (Figure 7A). PFA had two new peaks at 1500–400 cm^−1^, 827 cm^−1^ (characteristic of benzene ring para-substitution), and 1332 cm^−1^ (C–H symmetry deformation vibration of CH_3_). PV had a new peak at 812 cm^−1^ (stretching the vibration of the benzene ring). This showed that the interaction between FA/vitexin, and globulin produced new groups and substances. Kanakis et al. [53] believed that the influence of polyphenols on the protein structure was mainly due to an α-spiral, β-fold, β-corner, and irregular curl, which were caused by the conversion between the four structural contents. Table 3 shows the results obtained using Fourier deconvolution spectral fitting analysis of the Amide I band. Table 3 shows that the structures of the interaction products at different polyphenol concentrations change to different degrees. Compared with single-component mung bean globulin, with the increase in FA concentration, the β-fold and irregular curl content of PFA increased, the β-corner content was reduced, and the α-spiral content did not change significantly. The trend change in PV was consistent with that of PFA (Figure 7B). This showed that adding polyphenols could change the secondary structure of mung bean globulin, reduce its disordered structure, and make it more stable. The results were consistent with those of Yan et al. [54]. After heating, the α-spiral of PFA turned into a β-fold. Covalent bonds can promote the formation of protein structures that form β-fold more efficiently [55]. PFA showed a red shift in the Amide I and III bands. Heating could promote the covalent binding between FA and mung bean globulin (Figure 7C). After heating, the β-fold and irregular curl content of PV increased significantly, and the α-helix and β-fold content decreased significantly. Simultaneously, there was a blue shift phenomenon. The change in the α-spiral was related to the combination of hydrogen bonds and polyphenols in the hydrophobic region of the protein, and the decrease in the β-fold was related to the increase in protein site exposure after the folding of the protein hydrophobic region. The change in the characteristic structure made the protein highly disordered [54]. After heating, the structure of PFA was more stable than that of PV (Figure 7D).

### 3.6. SDS–PAGE 

**Figure 8:**
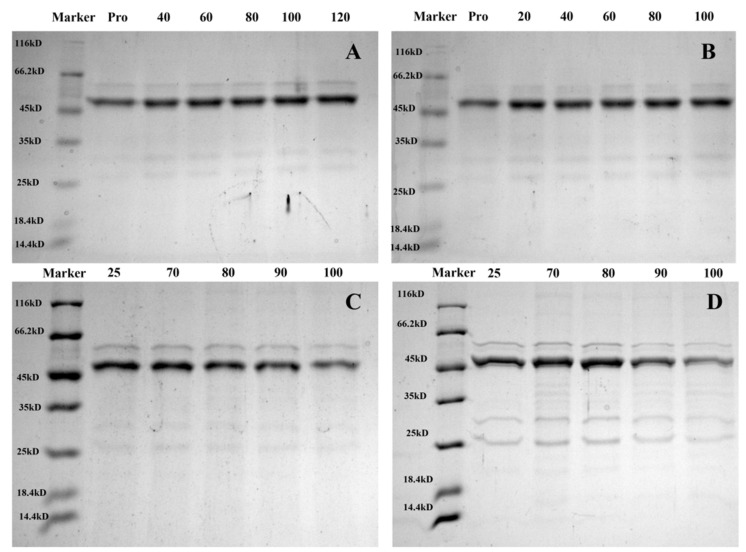
SDS–PAGE electrophoretogram: (**A**) Mung bean globulin–ferulic acid complex in different proportions; (**B**) Mung bean globulin–vitexin complex in different proportions; (**C**) Mung bean globulin–ferulic acid complex at different temperatures; (**D**) Mung bean globulin–vitexin complex at different temperatures.

Changes in the protein secondary structure could be observed using SDS–PAGE. Figure 8 shows that, with the increase in FA/vitexin concentration, the subunit bands with PFA and PV molecular weights of 59.7 kDa and 60.9 kDa, respectively, gradually darkened. The molecular weight showed that the degree of cross-linking between FA/vitexin and mung bean globulin gradually increased, and more macromolecular polymers were generated, which is conducive to the formation of more stable complexes [56]. The molecular weights of the two subunit bands of PFA and PV differed, which might be because the molecular weight of vitexin was greater than that of FA, resulting in the molecular weight of PV being greater than that of PFA. With increasing temperature, the PFA and PV subunit bands gradually became shallow, and a small amount of polymer conjugates was formed. This confirmed the results obtained by the above-infrared spectra. With increasing temperature, the two subunit bands with molecular weights of 31.4 kDa and 25.7 kDa of PV gradually became shallow. However, this was not the case for PFA. This might be due to the increase in temperature, which destroyed the binding of globulin and vitexin; globulin was decomposed into a small molecular protein and then recombined with vitexin, which might alter the charge around it [57], resulting in the gradual weakening of the band. Therefore, the PFA structure after heating was more stable than that of PV. Liu et al. [58] reported the same phenomenon in a kidney bean protein–polyphenol complex after heating, which is consistent with the results in this paper.

### 3.7. Particle Size and ζ Potential

**Figure 9:**
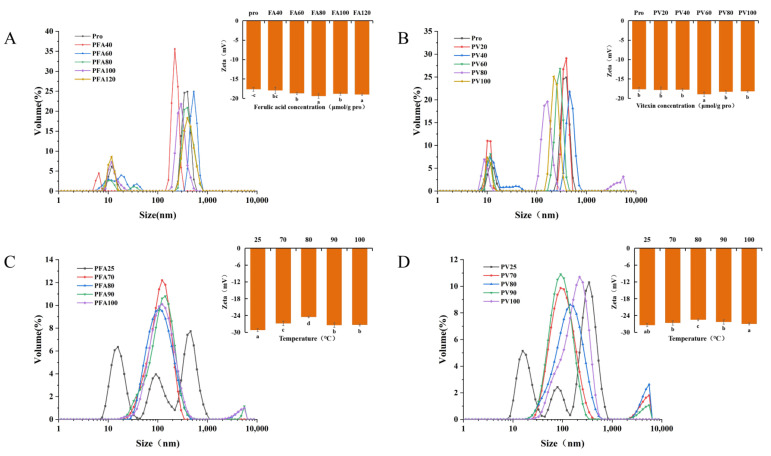
Particle size and ζ potential plots: (**A**) Mung bean globulin–ferulic acid complex in different proportions; (**B**) Mung bean globulin–vitexin complex in different proportions; (**C**) Mung bean globulin–ferulic acid complex at different temperatures; (**D**) Mung bean globulin–vitexin complex at different temperatures. Different letters within a picture indicate significant differences for each parameter (*p* < 0.05).

Figure 9 shows that with the addition of FA/vitexin, the particle size of the two compounds increased and then decreased. At the same concentration, the particle size of PFA was slightly larger than that of PV. This may be because the increase in polyphenol concentration reduced the pH value of the composite system. Mung bean globulin was positively charged away from the isoelectric point, and the surface charge of mung bean globulin increased, thereby enhancing the electrostatic repulsion of the complex, resulting in a gradual increase in particle size. With the increase in polyphenol concentration, the interaction produced a repulsive electric force, resulting in a smaller particle size [59]. With the increase in temperature, the particle size of the two composites showed a downward trend. After heat treatment, the hydrophobic group of globulin was exposed, the flexible structure of globulin was expanded, the surface charge was reduced, and the electrostatic repulsion was weakened. Therefore, the particle size of the two complexes was reduced. The particle size of PV after heat treatment was smaller than that of PFA, indicating that temperature had a greater influence on PV.

The greater the ζ potential, the greater the degree of mutual attraction between the composites, which further revealed that the stability of the composite was greater [60]. The ζ potential of mung bean globulin was negative, indicating that the negatively charged group on the surface of mung bean globulin was dominant. The addition of FA/vitexin increased the complex absolute value of the ζ potential, and the ζ potential of the absolute value of the potential of PV was greater than that of PFA. This might be due to the deprotonation of vitexin, which contained more phenolic hydroxyl groups under neutral conditions, making it negatively charged. After binding with globulin, it neutralized the positive charge on the protein surface, thereby increasing the negative charge on the protein surface [61]. After heat treatment, the ζ potential of the absolute value of the potential of PFA/PV decreased to different degrees, indicating that heat treatment could reduce the stability of PV and PFA. This was consistent with the infrared spectroscopy results. In addition, a difference in peak shape between the two compounds was observed. Compared with that of PFA, the peak of PV was narrower, indicating that the particle size distribution was more concentrated than that of PFA. After heating, the size distribution of PFA particles was relatively concentrated, and the relative dispersion distribution of composite particles may be due to the different covalence of the combined products. According to Zhou et al., the oxidation and cross-linking of polyphenols in the sample of a protein–polyphenol covalent complex may increase the particle size and the formation of a stable network structure [62]. Therefore, polymers with a larger particle size might be produced by combining polyphenols and proteins with various reactive groups.

### 3.8. Surface Hydrophobicity

**Figure 10:**
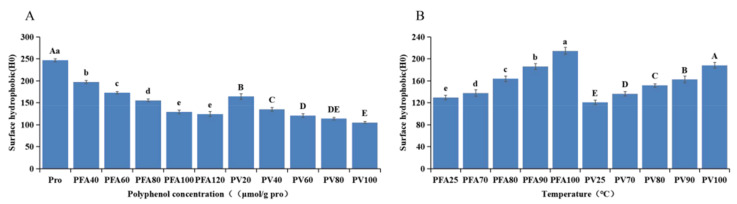
(**A**) Surface hydrophobicity of mung bean globulin after reaction with different concentrations of polyphenols; (**B**) Effect of heat treatment on surface hydrophobicity of the mung bean globulin–polyphenol complex. Note: Different letters indicate differences, and there were significant differences between the groups (*p* < 0.05).

Studies have shown that the concentration, type, and structure of polyphenols can change the secondary structure of the polyphenol–protein complex compared with that of natural protein [63]. The surface hydrophobicity of proteins decreased, indicating that the protein structure became loose and was more easily exposed to the solution [64]. As shown in Figure 10, compared with mung bean globulin, the surface hydrophobicity of the two complexes gradually decreased with the increase in FA/vitexin concentration (*p* < 0.05). This was related to the burial of hydrophobic amino acid residues in the binding protein during the interaction process [65]. Simultaneously, introducing hydrophilic amino acid residues from polyphenols reduces protein surface hydrophobicity [64]. In addition, at the same concentration, the surface hydrophobicity of PFA was lower than that of PV, which might be due to the electrostatic interaction in PV. Thus, retaining the α-spiral structure made its surface hydrophobicity relatively high [66]. However, after heat treatment, the surface hydrophobicity of the two complexes increased to varying degrees. After the temperature increased, the globulin molecules gradually expanded, and the hydrophobic groups on the surface of the globulin were exposed to the polar environment, resulting in a gradual increase in the surface hydrophobicity of the complex.

## 4. Conclusions

In this study, one phenolic acid (FA) and one flavonoid (vitexin) with different structures from mung beans were used to interact with mung bean globulin, and the changes in structure and antioxidant activity of the two compounds before and after heating were compared. The results showed that the antioxidant activity of PFA was significantly higher than that of PV at room temperature. Adding two kinds of polyphenols quenched the fluorescence of globulin, and PFA and PV bonded through electrostatic and hydrophobic interactions at one binding site. Furthermore, the interaction was a spontaneous reaction (∆G < 0), which changed the secondary structure of the protein. After the interaction between mung bean globulin and the two polyphenols, the particle size of mung bean globulin increased first and then decreased, and the cross-linking between the particles was formed. Meanwhile, surface hydrophobicity decreased significantly with the increase in the absolute value of potential. The increase in temperature significantly reduced the antioxidant activity of the compound and had a greater effect on PFA. The secondary structures changed, particle size decreased significantly, and surface hydrophobicity increased significantly. PFA was more stable than PV under heat treatment. The results of this study provide new possibilities for the development and utilization of mung bean globulin and mung bean polyphenols in food systems. Although temperature is an important factor affecting the interaction between mung bean protein and polyphenols, the influence of pH, ion concentration, and other factors should not be ignored. The influence of these factors on the mung bean globulin–polyphenol complex will be the focus of our future research.

## Figures and Tables

**Table 1:** Effect of heat treatment on the antioxidant activity of the mung bean globulin–polyphenol complex. Different letters within a column indicate significant differences for each parameter (*p* < 0.05).

Temperature (°C)	PV			PFA		
	DPPH (%)	ABTS (%)	FRAP (μmol TE/g)	DPPH (%)	ABTS (%)	FRAP (μmol TE/g)
25	64.04 ± 0.93 ^a^	83.13 ± 0.75 ^a^	72.13 ± 0.734 ^a^	77.29 ± 2.01 ^a^	82.69 ± 1.27 ^a^	80.46 ± 1.24 ^a^
70	52.53 ± 1.04 ^b^	73.66 ± 0.69 ^d^	40.56 ± 1.026 ^c^	52.14 ± 1.16 ^c^	74.18 ± 1.53 ^b^	59.27 ± 0.95 ^c^
80	47.17 ± 0.69 ^c^	76.34 ± 1.04 ^c^	45.72 ± 2.113 ^bc^	43.76 ± 0.96 ^d^	67.01 ± 0.94 ^d^	53.24 ± 0.87 ^d^
90	51.75 ± 1.43 ^b^	77.61 ± 2.078 ^b^	47.13 ± 1.314 ^b^	54.78 ± 1.04 ^bc^	70.52 ± 1.24 ^c^	61.33 ± 2.02 ^b^
100	52.73 ± 0.46 ^b^	78.28 ± 1.623 ^b^	48.66 ± 0.463 ^b^	57.6 ± 0.83 ^b^	71.87 ± 2.02 ^c^	62.55 ± 1.73 ^b^

**Table 2:** Fluorescence quenching constants, binding sites, and apparent binding constants of mung bean globulin–polyphenol complexes at different heat treatment temperatures.

Sample	T/K	K_SV_/(×10^3^ L/mol)	K_q_/[×10^12^/(mol·s)]	K_A_/(×10^4^ L/mol)	R^2^	n	ΔH/(kJ/mol)	ΔG/(kJ/mol)	ΔS/[J/(mol·K)]
PFA	298	4.21	4.21	4.19	0.9926	1.37	-	-	-
	343	2.61	2.61	32.97	0.9964	1.52	0.67	−2.64	47.25
	353	3.73	3.73	29.27	0.9973	1.48	0.59	−2.62	40.04
	363	1.89	1.89	19.40	0.9934	1.51	0.44	−2.53	33.02
	373	2.91	2.91	10.79	0.9955	1.38	0.26	−2.41	26.71
PV	298	36.40	36.40	499.80	0.9961	1.47	-	-	-
	343	5.86	5.86	251.65	0.9946	1.64	−0.22	−2.21	40.58
	353	6.80	6.80	150.18	0.9981	1.58	−0.36	−2.12	32.41
	363	5.06	5.06	520.60	0.9927	1.73	0.01	−3.21	35.85
	373	7.04	7.04	775.35	0.9956	1.78	0.12	−3.30	34.19

**Table 3:** Secondary structure composition of interactions between proteins and polyphenols of mung bean. Different letters within a column indicate significant differences for each parameter (*p* < 0.05).

Group	Sample	β-Sheet/%	α-Helix/%	Random Coil/%	β-Turn/%
	Pro	47.14 ± 0.12 ^Bc^	23.85 ± 0.12 ^Aa^	9.15 ± 0.17 ^Bc^	19.85 ± 0.11 ^Aa^
Ratio (μmol/g pro)	PFA40	49.15 ± 0.09 ^ab^	23.68 ± 0.08 ^ab^	10.71 ± 0.22 ^b^	16.46 ± 0.14 ^b^
	PFA60	49.31 ± 0.15 ^a^	23.66 ± 0.11 ^ab^	10.84 ± 0.26 ^b^	16.19 ± 0.10 ^bc^
	PFA80	49.12 ± 0.17 ^ab^	23.68 ± 0.19 ^ab^	10.69 ± 0.07 ^b^	16.51 ± 0.06 ^bc^
	PFA100	48.86 ± 0.06 ^b^	23.7 ± 0.15 ^a^	10.49 ± 0.15 ^b^	16.95 ± 0.17 ^b^
	PFA120	49.6 ± 0.23 ^a^	23.64 ± 0.24 ^b^	11.06 ± 0.18 ^a^	15.7 ± 0.21 ^c^
	PV20	49.44 ± 0.07 ^A^	23.65 ± 0.29 ^A^	10.94 ± 0.11 ^A^	15.96 ± 0.28 ^C^
	PV40	49.54 ± 0.16 ^A^	23.64 ± 0.18 ^A^	11.02 ± 0.09 ^A^	15.79 ± 0.14 ^C^
	PV60	49.48 ± 0.24 ^A^	23.82 ± 0.24 ^A^	9.42 ± 0.18 ^B^	17.28 ± 0.17 ^B^
	PV80	49.18 ± 0.19 ^A^	23.68 ± 0.16 ^A^	10.73 ± 0.21 ^A^	16.42 ± 0.16 ^BC^
	PV100	49.37 ± 0.27 ^A^	23.66 ± 0.19 ^A^	10.88 ± 0.16 ^A^	16.09 ± 0.23 ^C^
Temperature (°C)	PFA25	48.86 ± 0.13 ^d^	23.7 ± 0.14 ^a^	10.49 ± 0.23 ^a^	16.95 ± 0.22 ^bc^
	PFA70	49.97 ± 0.15 ^cd^	22.72 ± 0.16 ^bc^	9.04 ± 0.28 ^bc^	18.27 ± 0.14 ^a^
	PFA80	53.23 ± 0.26 ^a^	21.66 ± 0.23 ^c^	9.53 ± 0.19 ^b^	15.59 ± 0.09 ^c^
	PFA90	52.12 ± 0.21 ^b^	21.74 ± 0.05 ^c^	8.65 ± 0.16 ^c^	17.49 ± 0.26 ^b^
	PFA100	50.82 ± 0.08 ^c^	23.03 ± 0.11 ^ab^	10.69 ± 0.21 ^a^	15.47 ± 0.17 ^c^
	PV25	49.48 ± 0.24 ^A^	23.82 ± 0.24 ^A^	9.42 ± 0.18 ^B^	17.28 ± 0.17 ^C^
	PV70	47.18 ± 0.13 ^B^	20.98 ± 0.19 ^B^	10.23 ± 0.29 ^A^	21.6 ± 0.12 ^A^
	PV80	47.27 ± 0.24 ^B^	21.02 ± 0.24 ^B^	10.37 ± 0.14 ^A^	21.35 ± 0.25 ^A^
	PV90	47.19 ± 0.27 ^B^	20.99 ± 0.11 ^B^	10.25 ± 0.16 ^A^	21.56 ± 0.21 ^A^
	PV100	47.47 ± 0.09 ^B^	21.09 ± 0.17 ^B^	10.68 ± 0.19 ^A^	20.77 ± 0.08 ^B^

## Data Availability

The data presented in this study are available on request from the corresponding author.
